# Racial and Ethnic Disparities in Glycemic Control Among Insured US Adults

**DOI:** 10.1001/jamanetworkopen.2023.36307

**Published:** 2023-10-05

**Authors:** Nora I. Zakaria, Parisa Tehranifar, Blandine Laferrère, Sandra S. Albrecht

**Affiliations:** 1 Department of Epidemiology, Columbia University Mailman School of Public Health, New York, New York; 2 Department of Medicine, Division of Endocrinology, Diabetes Research Center, Columbia University Irving Medical Center, New York, New York

## Abstract

**Importance:**

Poor access to care and lack of health insurance are important contributors to disparities in glycemic control. However expanding health insurance coverage may not be enough to fully address the high burden of poor glycemic control for some groups.

**Objective:**

To characterize racial and ethnic disparities in glycemic control among adults with private and public insurance in the US over a 15-year timeframe and to evaluate whether social, health care, and behavioral or health status factors attenuate estimates of disparities.

**Design, Setting, and Participants:**

This cross-sectional study used data from the National Health and Nutrition Examination Survey from 2003 to 2018. Participants included Hispanic or Latino, non-Hispanic Black, and non-Hispanic White adults aged 25 to 80 years with self-reported diabetes and health insurance. Data were analyzed from January 15 to August 23, 2023.

**Exposure:**

Participants self-identified as Hispanic or Latino, non-Hispanic Black, or non-Hispanic White.

**Main Outcomes and Measures:**

The main outcome, poor glycemic control, was defined as glycated hemoglobin A_1c_ (HbA_1c_) of 7.0% or greater. Information about social (education, food security, and nativity), health care (insurance type, routine place for health care, insurance gap in past year, and use of diabetes medications), and behavioral or health status (years with diabetes, waist circumference, and smoking) factors were collected via questionnaires.

**Results:**

A total of 4070 individuals (weighted mean [SE] age, 61.4 [0.27] years; 1970 [weighted proportion, 49.3%] were women) were included, representing 16 337 362 US adults, including 1146 Hispanic or Latino individuals (weighted proportion, 13.2%), 1196 non-Hispanic Black individuals (weighted proportion, 15.7%), and 1728 non-Hispanic White individuals (weighted proportion, 71.1%). In models adjusted for age, sex, and survey year, Hispanic or Latino and non-Hispanic Black individuals had significantly higher odds of poor glycemic control than non-Hispanic White individuals (Hispanic or Latino: odds ratio [OR], 1.46; 95% CI, 1.16-1.83; Black: OR, 1.28; 95% CI, 1.04-1.57). There was some attenuation after adjustment for social factors, especially food security (Hispanic or Latino: OR, 1.39; 95% CI, 1.08-1.81); Black: OR, 1.39; 95% CI, 1.08-1.81). However, accounting for health care and behavioral or health status factors increased disparities, especially for Hispanic or Latino individuals (OR, 1.63; 95% CI, 1.24-2.16), with racial and ethnic disparities persisting even among those with private insurance (OR, 1.66; 95% CI, 1.10-2.52).

**Conclusions and Relevance:**

In this cross-sectional study of insured adults with diabetes in the US, disparities in poor glycemic control persisted despite adjustment for social, health care, and behavioral factors. Research is needed to identify the barriers contributing to poor control even in populations with access to care.

## Key Points

**Question:**

Do disparities in glycemic control associated with race and ethnicity persist over time among adults with private and public insurance and do social, health care, and behavioral or health factors attenuate disparity estimates?

**Findings:**

In this cross-sectional study of 4070 US adults, Hispanic or Latino and non-Hispanic Black individuals had higher odds of poor glycemic control compared with non-Hispanic White individuals, despite high levels of access to care. Social, health care, and behavioral or health factors did not attenuate disparities, which also persisted among adults with private insurance.

**Meaning:**

These findings suggest that having health insurance was insufficient to address disparities in glycemic control associated with race and ethnicity; research is needed to identify barriers preventing Hispanic and Latino and non-Hispanic Black communities from meeting diabetes management goals.

## Teaser

This cross-sectional study describes racial and ethnic disparities in glycemic control among adults with private and public insurance in the US over a 15-year timeframe and evaluates whether social, health care, and behavioral or health status factors attenuate estimates of disparities.

## Introduction

Approximately 37.3 million individuals in the US have diabetes, and it is the seventh leading cause of death and the leading cause of comorbidities, including cardiovascular disease, amputation, and kidney failure, in the US.^1^ A crucial component of diabetes care is glycemic control, managed through medication, healthy diet, and physical activity.

US trends in glycemic control point to a recent pattern of decline. After some improvement from 1988 to 1994 through 2005 to 2010, the prevalence of glycemic control decreased from 57.4% of adults in 2007 to 2010 to 50.5% of adults in 2015 to 2018, based on data from the National Health and Nutrition Examination Surveys (NHANES).^2^ Across a similar timeframe, Hispanic or Latino and non-Hispanic Black adults bore a disproportionately higher burden of poor control compared with non-Hispanic White adults.^2,3,4^

Poor access to care and lack of health insurance are important contributors to disparities in glycemic control.^5^ Data from the 2020 National Health Interview Survey indicated that close to one-fourth of Hispanic or Latino adults younger than 65 years were uninsured, the highest of any racial or ethnic group.^6^ Health insurance is a critical component of diabetes care because coverage is associated with earlier detection and better management.^7,8^ The passage of the 2010 Patient Protection and Affordable Care Act expanded access to health insurance, narrowing gaps in coverage by race and ethnicity, but disparities persist.^9^

Despite the importance of having health insurance for the successful management of diabetes, there is some evidence that disparities in access to care do not fully account for the high burden of poor control among Hispanic or Latino and non-Hispanic Black individuals.^4,10^ According to Healthy People 2020,^11^ a *health disparity* is a health difference between groups that is unfair. For insured adults with diabetes, financial hardships can make it difficult to adhere to medications to achieve care goals. These and other barriers to accessing nutritious food can interfere with an individual’s ability to make healthy changes to diet.^12^ Other determinants of poor glycemic control, such as advanced disease at diagnosis, can be impacted by health care–related factors, such as gaps in health insurance coverage or disparities in access to screening.

Identifying the factors that are associated with poor diabetes management is essential for more effective care planning, especially for racial and ethnic groups that bear a disproportionate burden of diabetes-associated morbidity and mortality. Consistent with existing frameworks for addressing health disparities,^11,13,14^ this study does not focus on the causal status of race or ethnicity. Instead, we apply a descriptive approach to evaluate whether a range of potentially modifiable factors attenuate estimates of disparities in glycemic control. We used nationally representative data to evaluate trends in racial and ethnic disparities in glycemic control among US adults with private and public insurance over a 15-year timeframe. We then evaluated whether a range of social, health care, and behavioral or health status factors attenuated estimates of disparities.

## Methods

This cross-sectional study was approved by the National Center for Health Statistics (NCHS) institutional review board approved the surveys, and all participants provided informed consent. This study followed the Strengthening the Reporting of Observational Studies in Epidemiology (STROBE) reporting guideline for cross-sectional studies.

Data came from NHANES, a series of ongoing national surveys conducted by the NCHS. NCHS has conducted surveys in 2-year cycles since 1999. NHANES uses a complex, multistage sample design intended to be nationally representative of the US noninstitutionalized population.^15^ In the first phase, researchers collected information from household interviews on demographics, socioeconomic indicators, medical history, and health behaviors. In the second phase, participants were administered a physical examination in a mobile examination center.

We pooled 15 years of data from 2003 to 2018 to achieve sufficient sample sizes. We restricted the sample to adults aged 25 to 80 years with diabetes who reported having health insurance, self-reported their race and ethnicity as Hispanic or Latino (combines the Mexican American and Other Hispanic racial and ethnicity categories), non-Hispanic Black, or non-Hispanic White and had complete data on hemoglobin A_1c_ (HbA_1c_). We did not include participants self-reporting other race (including multiracial) due to small sample size. Participants were classified as having diabetes if they reported having had a physician’s diagnosis of diabetes (other than during pregnancy). HbA_1c_ was measured using whole blood at a central laboratory by a high-performance liquid chromatographic assay and standardized according to the Diabetes Control and Complications method.^15^ Poor glycemic control was defined by HbA_1c_ levels 7.0% or greater (to convert to proportion of total hemoglobin, multiply by 0.01), consistent with other population-based studies.^2,3,10^ In sensitivity analyses, we repeated analyses using less stringent targets: 8.0% or greater and 9.0% or greater.

### Covariates

Information on other demographic, social, health care, and behavioral or health status factors was collected during the home interview. Social factors included nativity (US-born or non–US-born), education (<high school, high school, some college, or ≥college), and food security, assessed via the US Department of Agriculture 18-item household questionnaire (full food security, marginal food security, low food security, and very low food security).^16^ Health care–related factors included diabetes medication use (insulin only, oral hypoglycemic agents only, insulin and oral hypoglycemic agents, or none), having a routine place for health care (yes or no), having insurance gaps in the past year (yes or no), and health insurance type. Although NHANES captures various insurance types, we categorized this variable as private, Medicare or Medicaid, and other due to small sample sizes for other insurance types. We considered evaluating what an individual’s health care professional suggested their A_1c_ should be, but these data were not available across all years, and among the years in which these data were collected, missingness was disproportionately high, especially for Hispanic or Latino participants (approximately 33%) and non-Hispanic Black individuals (approximately 26%) compared with non-Hispanic White individuals (approximately 15%). Behavioral or health status factors included years with diabetes, waist circumference, and smoking status (never, former, or current). Waist circumference was measured in centimeters at the midpoint between the bottom of the ribs and the top of the iliac crest. For descriptive purposes, we also dichotomized waist circumference based on established criteria for defining abdominal obesity.^17^ We chose to evaluate waist circumference over body mass index because several studies have shown that waist circumference is a better indicator of metabolic health.^18^ Higher waist circumference has also been associated with with increased sedentary activity and diets high in sugar and energy-dense foods.^19^

### Statistical Analysis

All analyses were conducted using Stata statistical software version 14.0 (StataCorp). Survey procedures were used to account for the complex design. Appropriate weights were applied to provide unbiased population estimates and account for survey nonresponse, oversampling, poststratification, and sampling error.^20^ Weighted proportions and means were used to characterize the analytic sample by race and ethnicity. Differences were evaluated using the *t* statistic, and 2-sided *P* < .05 was considered statistically significant. Multiple imputation with chained equations was used to deal with missing data for covariates (approximately 12% missing). Based on previous recommendations, 20 imputations were used.^21^ Multivariable logistic regression models were used to assess the associations of race and ethnicity with poor glycemic control, defined as HbA_1c_ 7.0% or greater, adjusting for age, sex, and survey year (model 1). We next added the social, health care, and behavioral or health status variables to the model in a sequential manner to evaluate the degree to which estimates of racial and ethnic disparities were attenuated as they were conditioned on each new set of variables. Model 2 further adjusted for nativity, education, and food security. Model 3 further adjusted for diabetes medication use, having a routine place for health care, having insurance gaps in the past year, and insurance type. Model 4 included additional adjustments for years with diabetes, waist circumference, and smoking status. Marginal effects were also calculated for the race and ethnic variables at the sample mean of the covariates. In sensitivity analyses, we evaluated whether disparities persisted by health insurance type (private vs Medicare or Medicaid) using stratified models. We did not stratify on other insurance type due to small sample size. We also reran all models using glycemic targets of 8.0% or greater and 9.0% or greater. Data were analyzed from January 15 to August 23, 2023.

## Results

A total of 4070 adults (weighted mean [SE] age, 61.4 [0.27] years; 1970 [weighted proportion, 49.3%] female) were included in this study, representing 16 337 362 insured adults with diagnosed diabetes in the US. Table 1 displays characteristics by race and ethnicity. There were 1146 Hispanic or Latino individuals (weighted proportion, 13.2%), 1196 non-Hispanic Black individuals (weighted proportion, 15.7%), and 1728 non-Hispanic White individuals (weighted proportion, 71.1%). Compared with non-Hispanic White individuals, a higher proportion of Hispanic or Latino and Black individuals had less than a high school education and were less food secure (Table 1). A small proportion of non-Hispanic Black and non-Hispanic White individuals were born outside the US, whereas most Hispanic or Latino individuals were born outside the US (Table 1). Although private insurance was the most common insurance type across racial and ethnic groups, a higher proportion of Hispanic or Latino and non-Hispanic Black individuals had Medicare or Medicaid. Most of the population, regardless of race or ethnicity, had a routine place for health care, but estimates were lower for Hispanic or Latino individuals compared with non-Hispanic White individuals (Table 1). There were no statistically significant differences by race or ethnicity in the proportion of adults with past-year gaps in insurance, in the proportion using diabetes medications, nor in the number of years with diabetes. Hispanic or Latino individuals had the lowest proportion of individuals with high waist circumference, and Hispanic or Latino and non-Hispanic Black individuals had higher proportions of adults who never smoked compared with White individuals (Table 1).

**Table 1.** Characteristics of Insured Adults Aged 25 to 80 Years With Diagnosed Diabetes by Race and Ethnicity^a^

Characteristic	Individuals, unweighted No. (weighted %)			*P* value
	Hispanic or Latino (n = 1146)	Non-Hispanic Black (n = 1196)	Non-Hispanic White (n = 1728)	
Age (SE), y	57.8 (0.62)	59.5 (0.47)	62.5 (0.36)	<.001
Sex				
Male	571 (49.5)	578 (42.3)	951 (52.8)	<.001
Female	575 (50.5)	618 (57.7)	777 (47.2)	
Highest educational attainment, unweighted No. (weighted %)				
<High school	630 (47.8)	366 (28.3)	422 (17.0)	<.001
High school graduate or GED	201 (19.3)	290 (25.1)	464 (26.8)	
Some college	232 (25.0)	363 (31.5)	517 (32.7)	
≥College graduate	80 (7.8)	172 (15.1)	325 (23.5)	
Food security				
Full	670 (57.7)	766 (63.7)	1298 (81.7)	<.001
Marginal	144 (13.9)	165 (15.0)	143 (7.6)	
Low	201 (17.7)	152 (13.3)	156 (6.6)	
Very low	104 (10.7)	95 (8.0)	102 (4.1)	
Nativity				
US-born	494 (44.2)	1099 (92.2)	1664 (96.7)	<.001
Non–US-born	652 (55.8)	97 (7.8)	64 (3.3)	
Insurance type				
Private insurance	475 (49.2)	598 (52.6)	992 (66.8)	<.001
Medicare or Medicaid	556 (40.8)	496 (39.0)	621 (26.2)	
Other	111 (1.0)	97 (8.4)	110 (7.0)	
Have routine place for health care	1102 (95.5)	1179 (98.2)	1696 (97.8)	.04
Time with no insurance in past year				
Yes	250 (21.0)	168 (15.8)	200 (15.3)	.20
No	844 (74.1)	988 (80.1)	1479 (82.0)	
Missing	52 (4.9)	40 (4.0)	49 (2.8)	
Using diabetes medication				
Insulin only	128 (11.6)	192 (15.9)	254 (14.6)	.29
Oral hypoglycemic agents only	667 (56.1)	671 (55.0)	964 (56.6)	
Insulin and oral hypoglycemic agents	188 (15.5)	185 (15.7)	264 (14.6)	
No medications	163 (16.8)	148 (13.4)	246 (14.1)	
Diabetes duration, mean (SE), y	11.9 (0.42)	12.0 (0.33)	12.3 (0.36	.77
Waist circumference				
Mean (SE), cm	108.0 (0.61)	111.6 (0.56)	112.7 (0.50)	<.001
High-risk^b^	825 (71.7)	886 (75.3)	1342 (79.7)	<.001
Smoking status				
Current smoker	135 (13.0)	220 (18.5)	251 (13.7)	<.001
Former smoker	393 (31.8)	398 (30.2)	727 (39.7)	
Never smoker	618 (55.2)	576 (51.3)	750 (46.6)	

Abbreviation: GED, general equivalency diploma.

^a^ Diabetes diagnosis was based on self-report of a previous diagnosis by a physician or other health professional.

^b^ Defined as waist circumference greater than 102 cm for men and greater than 88 cm for women.

Racial and ethnic differences in glycemic control persisted across the 15-year period among insured adults (Figure), including in more recent years (2015-2018), after the implementation of the 2010 Patient Protection and Affordable Care Act. Across all years, the prevalence of poor glycemic control was highest among Hispanic or Latino individuals, followed by non-Hispanic Black and non-Hispanic White individuals.

**Figure.**
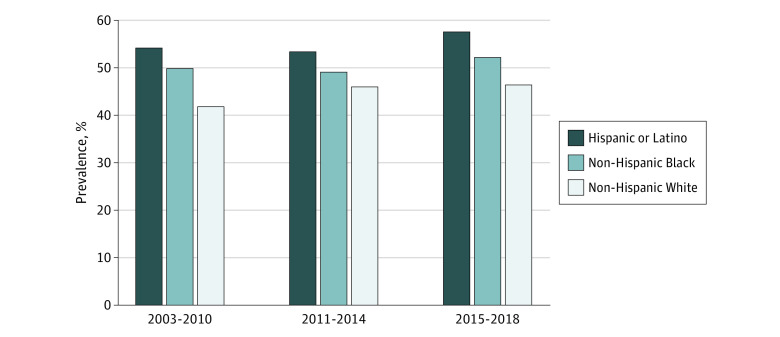
Prevalence of Poor Glycemic Control Among Insured Adults With Diabetes by Race and Ethnicity Poor glycemic control was defined as glycated hemoglobin A_1c_ 7.0% or greater (to convert to proportion of total hemoglobin, multiply by 0.01).

Table 2 presents results from multivariable regressions combining years 2003 to 2018. In analyses adjusted for age, sex, and survey year (model 1), Hispanic or Latino individuals (odds ratio [OR], 1.46; 95% CI, 1.16-1.83) and non-Hispanic Black individuals (OR, 1.28; 95% CI, 1.04-1.57) had significantly higher odds of poor glycemic control compared with non-Hispanic White individuals. There was some attenuation of estimates after adjusting for the disproportionate burden of low education, immigrant status, and low food security among Hispanic or Latino (OR, 1.39; 95% CI, 1.08-1.81) and non-Hispanic Black (OR, 1.39; 95% CI, 1.08-1.81) individuals (model 2). Food security contributed most to the attenuation (noted when adjusting for each variable individually in sensitivity analyses). Nevertheless, estimates remained statistically significant, especially for Hispanic or Latino individuals. Further adjustment for health care–related factors (model 3) resulted in an even larger magnitude of association. In sensitivity analyses, we evaluated the impact of each health care–related variable on associations to understand which variables were more or less influential in inflating estimates of disparities. Adjusting for the use of diabetes medications contributed most to the widening of disparities, especially for Hispanic or Latino individuals. When we stratified models on medication use, disparities were evident across all strata, but were especially large among adults not using diabetes medication and among individuals using both insulin and oral hypoglycemic agents. Accounting for the lower waist circumference and more favorable smoking status for Hispanic or Latino and non-Hispanic Black individuals, in addition to years with diabetes (model 4), increased estimates of racial and ethnic disparities in probability of poor glycemic control of 62.0% (95% CI, 52.3%-71.6%) for Hispanic or Latino individuals, 56.6% (95% CI, 47.1%-66.0%) for non-Hispanic Black individuals, and 49.9% (95% CI, 40.3%-59.6%) for non-Hispanic White individuals.

**Table 2.** Multivariable Logistic Regression of Poor Glycemic Control by Race and Ethnicity

Characteristic	OR (95% CI)^a^			
	Model 1	Model 2	Model 3	Model 4
Race and ethnicity				
Hispanic or Latino	1.45 (1.16-1.82)	1.39 (1.08-1.81)	1.58 (1.20-2.09)	1.63 (1.24-2.16)^b^
Non-Hispanic Black	1.27 (1.03-1.56)	1.24 (1.01-1.52)	1.29 (1.05-1.60)	1.30 (1.05-1.62)^b^
Non-Hispanic White	1 [Reference]	1 [Reference]	1 [Reference]	1 [Reference]^b^
Age, per 1-y increase	0.99 (0.98-0.99)	0.99 (0.98-0.99)	0.99 (0.98-0.99)	0.99 (0.98-0.99)
Sex				
Male	1.33 (1.14-1.56)	1.36 (1.16-1.60)	1.34 (1.12-1.61)	1.33 (1.10-1.61)
Female	1 [Reference]	1 [Reference]	1 [Reference]	1 [Reference]
Nativity				
Non–US-born	NA	1.01 (0.78-1.30)	1.112 (0.84-1.46)	1.20 (0.91-1.59)
US-born	NA	1 [Reference]	1 [Reference]	1 [Reference]
Education				
<High school	NA	1 [Reference]	1 [Reference]	1 [Reference]
High school graduate or GED	NA	1.16 (0.92-1.46)	1.13 (0.88-1.45)	1.17 (0.92-1.50)
Some college	NA	0.94 (0.72-1.22)	0.99 (0.73-1.35)	1.01 (0.75-1.38)
≥College graduate	NA	0.94 (0.72-1.22)	0.94 (0.69-1.27)	1.00 (0.74,1.34)
Food security				
Very low	NA	1.37 (0.99-1.90)	1.15 (0.77-1.74)	1.11 (0.74-1.67)
Low	NA	1.12 (0.83-1.51)	1.11 (0.81-1.52)	1.07 (0.78-1.47)
Marginal	NA	1.05 (0.79-1.40)	0.93 (0.71-1.23)	0.91 (0.68-1.20)
Full	NA	1 [Reference]	1 [Reference]	1 [Reference]
Insurance type				
Medicare or Medicaid	NA	NA	0.83 (0.67-1.04)	0.82 (0.66-1.02)
Other	NA	NA	0.72 (0.50-1.04)	0.71 (0.50-1.01)
Private	NA	NA	1 [Reference]	1 [Reference]
Routine place for health care				
Yes	NA	NA	0.55 (0.26-1.16)	0.56 (0.28-1.13)
No	NA	NA	1 [Reference]	1 [Reference]
Gap in insurance in past 1 y				
Yes	NA	NA	1.64 (1.03-2.60)	1.63 (1.03-2.59)
No	NA	NA	1 [Reference]	1 [Reference]
Missing	NA	NA	0.97 (0.59-1.58)	0.99 (0.60-1.64)
Diabetes medication				
Insulin only	NA	NA	12.89 (8.78-18.94)	12.00 (8.11-17.76)
Oral hypoglycemic agents only	NA	NA	3.67 (2.70-5.00)	3.58 (2.60-4.93)
Insulin and oral hypoglycemic agents	NA	NA	16.42 (11.36-23.74)	14.95 (10.18–21.96)
None	NA	NA	1 [Reference]	1 [Reference]
Waist circumference, per 1-cm increase	NA	NA	NA	1.01 (1.00-1.02)
Diabetes duration, per 1-y increase	NA	NA	NA	1.01 (1.00-1.02)
Smoking status				
Current smoker	NA	NA	NA	1.28 (0.96-1.71)
Former smoker	NA	NA	NA	0.91 (0.74-1.11)
Never smoker	NA	NA	NA	1 [Reference]

Abbreviations: GED, general equivalency diploma; NA, not applicable; OR, odds ratio.

^a^ All models include adjustment for survey year.

^b^ Corresponds to probabilities of poor glycemic control of 62.0% (95% CI, 52.3%-71.6%) for Hispanic or Latino individuals, 56.6% (95% CI, 47.1%-66.0%) for non-Hispanic Black individuals, and 49.9% (95% CI, 40.3%-59.6%) for non-Hispanic White individuals.

When we stratified models by health insurance type, disparities persisted for Hispanic or Latino individuals with private insurance (OR, 1.66; 95% CI, 1.10-2.52) and non-Hispanic Black individuals with Medicare or Medicaid (OR, 1.39; 95% CI, 1.02-1.88) compared with White individuals (Table 3). All the patterns we reported persisted when we evaluated other thresholds of glycemic control (Table 4).

**Table 3.** Multivariable Logistic Regression of Poor Glycemic Control by Race and Ethnicity, Stratified by Health Insurance Type

Race and ethnicity	OR (95% CI)^a^	
	Private insurance	Medicare or Medicaid
Hispanic or Latino	1.66 (1.10-2.52)	1.42 (0.97-2.09)
Non-Hispanic Black	1.26 (0.96-1.66)	1.39 (1.02-1.88)
Non-Hispanic White	1 [Reference]	1 [Reference]

Abbreviation: OR, odds ratio.

^a^ Models adjusted for age, sex, social, health care, and behavioral or health status factors and survey year. Stratified models of other insurance are not shown due to small sample size.

**Table 4.** Multivariable Logistic Regression of Poor Glycemic Control Across Different HbA_1c_ Cut Points, by Race and Ethnicity

Race and ethnicity	OR (95% CI)			
	Model 1^a^	Model 2^b^	Model 3^c^	Model 4^d^
**HbA_1c_ >8.0%**				
Hispanic or Latino	1.80 (1.43-2.27)	1.70 (1.25-2.31)	1.93 (1.40-2.66)	1.95 (1.42-2.70)
Non-Hispanic Black	1.57 (1.26-1.96)	1.50 (1.21-1.87)	1.60 (1.27-2.01)	1.58 (1.25-2.00)
Non-Hispanic White	1 [Reference]	1 [Reference]	1 [Reference]	1 [Reference]
**HbA_1c_ >9.0%**				
Race/ethnicity				
Hispanic or Latino	2.07 (1.60-2.69)	2.15 (1.55-2.97)	2.35 (1.68-3.27)	2.35 (1.68-3.27)
Non-Hispanic Black	1.92 (1.48-2.50)	1.89 (1.46-2.44)	1.98 (1.49-2.62)	1.93 (1.46-2.55)
Non-Hispanic White	1 [Reference]	1 [Reference]	1 [Reference]	1 [Reference]

Abbreviations: HbA_1c_, glycated hemoglobin A_1c_; OR, odds ratio.

SI conversion factor: To convert HbA_1c_ to proportion of total hemoglobin, multiply by 0.01.

^a^ Model 1 adjusts for age, sex, and survey year.

^b^ Model 2 adjusts for factors in model 1 plus social factors.

^c^ Model 3 adjusts for factors in model 2 plus health care factors.

^d^ Model 4 adjusts for factors in model 3 plus behavioral or health status factors.

## Discussion

In this cross-sectional study of nationally representative US data from 2003 to 2018, racial and ethnic disparities in poor glycemic control persisted in the US in a population with health insurance, and despite a high prevalence of having a routine health care practitioner. Hispanic and Latino individuals had the highest odds of poor control. There was some attenuation of estimates after adjustment for social factors previously associated with poor chronic disease management,^3^ particularly food security, but disparities persisted. Adjustment for health care–related factors, especially use of diabetes medications, and for behavioral or health status factors resulted in a widening of estimates of disparities. Similar patterns were found even among adults with private insurance, as well as those with Medicare or Medicaid. These findings suggest that expanding health insurance coverage alone is unlikely to fully address racial and ethnic disparities in glycemic control.

Previous studies have reported somewhat similar patterns. Using earlier NHANES survey years (1988-1994 and 1999-2002), researchers have reported persistence in racial and ethnic disparities in glycemic control after adjusting for access to health care.^10,22^ A study by Trivedi et al^23^ used data from Medicare managed-care plans from 1997 to 2003 and found that although there were improvements in several quality of care measures for both Black and White beneficiaries, the magnitude of the racial disparity in glycemic control increased over time.^23^ Similar to our findings, adjustment for socioeconomic variables did not account for this disparity,^10,22,23^ although most studies did not account for the range of factors considered in our study.

Building on past research in this area, we used nationally representative data to investigate whether various social, health care, and behavioral or health status factors could attenuate estimates of racial and ethnic disparities in glycemic control in a population of insured adults with diabetes. Somewhat unexpectedly, these factors did not attenuate disparities and, in some instances, resulted in a widening of the racial and ethnic gap. In terms of the social factors we considered, although Hispanic or Latino individuals and non-Hispanic Black individuals were disproportionately burdened by lower levels of educational and less food security than non-Hispanic White individuals, accounting for this uneven distribution resulted in only some attenuation of estimates. Socioeconomic status is a multidimensional construct that is challenging to measure, with a high potential for residual confounding.^24^ Measures of socioeconomic status, such as education, may be insufficient to capture the socioeconomic barriers to glycemic control. Previous research has also noted the logistical and financial burden of frequent physicians’ appointments and a chronic need for glucose-lowering medications to adequately manage diabetes, all of which pose challenges to low-income populations.^23,25^ This suggests that these more traditional social measures may be insufficient for capturing all the challenges patients face in managing diabetes.

Accounting for health care–related factors resulted in a widening of racial and ethnic disparities, especially for Hispanic or Latino individuals, and this was largely driven by adjustment for use of diabetes medications. The disparity was especially large among individuals not using medication, and also among those using both insulin and oral hypoglycemic agents. Unmeasured socioeconomic factors could play a role. Cost-related nonadherence to medications is exemplified by delaying therapy or taking smaller or less frequent doses and has been shown to increase the risk of avoidable complications for individuals with chronic diseases, including diabetes,^25,26^ and to disproportionately impact Hispanic or Latino and non-Hispanic Black individuals.^27,28^ Unfortunately, we did not have data on cost-related nonadherence or other factors (eg, pharmacy accessibility, length of time using medications, other reasons for nonadherence) that may provide more insight. Future research should examine the reasons for these patterns.

We also observed that accounting for years with diabetes, waist circumference, and smoking status further magnified racial and ethnic disparities in glycemic control. Unlike the adverse patterning seen for the social indicators, Hispanic or Latino and non-Hispanic Black individuals had more favorable profiles than non-Hispanic White individuals regarding smoking status. Moreover, Hispanic or Latino individuals had the lowest proportion of adults with high waist circumference. However, once we adjusted for these theoretically protective factors among Hispanic or Latino and Black individuals, disparities in glycemic control widened. This suggests that finding ways to support the maintenance of these healthier profiles may be one component of a broader strategy to address disparities.

We also demonstrated that disparities persisted regardless of insurance type and were especially large among Hispanic or Latino individuals compared with non-Hispanic White individuals with private health insurance. Although this may seem counterintuitive, private insurance is often accompanied by high deductibles, copays, and coinsurance, which can pose great financial burden. Indeed, high-deductible private insurance plans have been previously associated with forgoing care among lower-income adults with diabetes, which was in turn associated with poor management outcomes.^29^ A recent analysis of adults with employer-sponsored health insurance also revealed concerning racial and ethnic disparities across a range of chronic disease outcomes, including diabetes.^30^ These findings suggest that barriers related to quality and processes of care faced by Hispanic or Latino and non-Hispanic Black individuals may be contributing to these patterns.

### Limitations

Our study has some limitations. NHANES data are cross-sectional; therefore, it is not possible to assess whether the factors we assessed had any causal association with glycemic control. It is also possible that the cumulative impacts of some factors (eg, poverty, food insecurity, years with health insurance) are more impactful than the 1-time measurement available in NHANES. We also could not distinguish diabetes by type. Treatment guidelines differ by type, and it would have been useful to make this distinction. We also did not have information surrounding treatment adherence, which is known to impact glycemic outcomes. Having this information would allow for improved targeting of diabetes management strategies. We also could not adequately assess the role of health care practitioner glycemic recommendations, since this information was not consistently collected across all the years included in this analysis, and because of the large degree of missingness. However, we noted that missingness disproportionately impacted Hispanic or Latino and non-Hispanic Black individuals. If we assume that missingness correlates with a lack of knowledge of an individual’s HbA_1c_ goals, this highlights a need to understand whether health care practitioners are adequately communicating this information to their patients. Furthermore, although we accounted for the length of time with diabetes, existing evidence has documented racial and ethnic disparities in undiagnosed diabetes.^31,32,33^ Although there were no racial and ethnic differences in years with diabetes, Hispanic or Latino and non-Hispanic Black adults could have been living with diabetes for more time than non-Hispanic White adults, potentially leading to more severe disease, rendering it more difficult to control.

## Conclusions

This cross-sectional study found persistent racial and ethnic disparities in poor glycemic control, even among adults with health insurance. These findings suggest that although improving access to health care remains a critical policy target, health insurance coverage alone was not enough to reduce disparities in diabetes management. Hispanic and Latino and non-Hispanic Black individuals experience a disproportionate burden of diabetes-related morbidity and mortality and associated costs.^34^ Uncontrolled diabetes has also been linked to more severe consequences from COVID-19.^35^ The social, health care, and behavioral or health status factors we examined did not attenuate estimates of disparities. Future studies should apply causal frameworks to evaluate the role of other structural barriers contributing to the high burden of poor control among insured Hispanic or Latino and non-Hispanic Black individuals to develop effective interventions.

## Notes

Supplement.
Data Sharing Statement
