# Supplementary material for: Racial and Ethnic Disparities in Glycemic Control Among Insured US Adults
Source: JAMA Netw Open. 2023 Oct 5;6(10):e2336307. doi: 10.1001/jamanetworkopen.2023.36307 (PMC10556965; doi:10.1001/jamanetworkopen.2023.36307)
Supplement: Supplement. — Data Sharing Statement [file jamanetwopen-e2336307-s001.pdf]

Data Sharing Statement

Zakaria. Racial and Ethnic Disparities in Glycemic Control Among Insured US Adults. *JAMA Netw Open*. Published October 05, 2023. doi:10.1001/jamanetworkopen.2023.36307

Data
Data available: Yes
Data types: Deidentified participant data
How to access data: The NHANES database is publicly accessible and contains deidentified data for all participants utilized in this study. This dataset can be accessed, queried, and downloaded using this link: https://wwwn.cdc.gov/nchs/nhanes/Default.aspx
When available: With publication

Supporting Documents
Document types: None

Additional Information
Who can access the data: Since NHANES is public, the data are always available.
Types of analyses: Since NHANES is public, the data are always available.
Mechanisms of data availability: The data are always publicly accessible and can be accessed at the NHANES website any time.
Any additional restrictions: None
